# Changes in patients’ outlook, behaviors, and attitudes toward COVID-19 after hospitalization and their experiences of discrimination and harassment

**DOI:** 10.1186/s13104-021-05780-9

**Published:** 2021-09-17

**Authors:** Ken Goda, Tsuneaki Kenzaka, Shinsuke Yahata, Ayako Kumabe, Masahiro Katsurada, Hogara Nishisaki

**Affiliations:** 1 Division of Community Medicine and Career Development, Kobe University Graduate School of Medicine, 2-1-5, Arata-cho, Hyogo-ku, Kobe, Hyogo 652-0032 Japan; 2 Department of Internal Medicine, Hyogo Prefectural Tamba Medical Center, Tanba, Japan; 3 Division of Community Medicine and Medical Education, Kobe University Graduate School of Medicine, Kobe, Japan; 4 Department of Respiratory Medicine, Division of Internal Medicine, Kobe University Graduate School of Medicine, Kobe, Japan

**Keywords:** COVID-19, Stigma, Pandemics, Presenteeism, Social support

## Abstract

**Objective:**

This study aims to examine changes in patients’ perspectives and outlooks regarding the disease and their health after hospitalization for COVID-19 and investigate their discrimination and harassment experiences. This prospective observational study surveyed discharged patients who had been admitted to Hyogo Prefectural Tamba Medical Center in Japan for COVID-19. Patient characteristics, changes in outlook and behaviors after discharge, and incidents of discrimination and harassment were examined. The study was conducted in two waves: March–June 2020 and July–September 2020.

**Results:**

Responses were obtained from 27 patients aged 50 ± 17 years, including 16 men (59.3%). We found most patients feared infection before hospitalization (88.5%) and had taken some preventive measures (96.3%), however after discharge, all (100%) practiced social distancing and infection prevention. Twenty patients (80%) considered changing their lifestyles, and 19 (79.2%) decided to use sick leave when they felt ill; these trends were more prominent during the second wave. Six patients (23.1%) reported experiencing discrimination or harassment after discharge. While most patients with COVID-19 had a strong fear of infection before hospitalization, their views about health and health behaviors changed after hospitalization.

**Supplementary Information:**

The online version contains supplementary material available at 10.1186/s13104-021-05780-9.

## Introduction

The novel coronavirus disease (COVID-19) outbreak is a global issue, and COVID-19 has been identified as a risk factor for mental health [1]. These unpredictable and uncertain circumstances have caused social isolation due to lockdowns and physical distancing, decreased income, loneliness, decreased activities, and restricted mobility, raising concerns regarding the impact of increased drinking and gambling and decreased family or social support—particularly for older adults [1]. Since it is assumed that both asymptomatic and pre-symptomatic individuals may transmit the virus [2], many patients are forced to be hospitalized even if their conditions are too mild to necessitate hospitalization under ordinary circumstances [2]. In addition to being a risk factor for mental health problems, this disease is associated with changes in everyday environments, such as home or work, hospitalization, discrimination, and harassment.

The impacts of COVID-19 are a concern [3] and some reports describe changes in mental health, well-being, and views on health for patients who contracted COVID-19. There have been reports of patients experiencing discrimination and harassment [4, 5]. However, no comprehensive studies exist on such changes or experiences among patients hospitalized with COVID-19 in Japan.

The present study aimed to investigate the conditions affecting perceptions and behaviors related to COVID-19 among patients hospitalized with the disease, the effects on their well-being, and their views on health before and after hospitalization. We also sought to document any patient’s experiences of discrimination and harassment related to having COVID-19.

## Main text

### Method

The present study is a prospective observational study, approved by the ethics committee of Hyogo Prefectural Tamba Medical Center (approval number: Tan-I number 1050). Written informed consent was obtained from patients. The participants were adult patients admitted to Hyogo Prefectural Tamba Medical Center for COVID-19 infection from March to September 2020.

At the time of discharge, questionnaire forms were distributed to the patients to survey changes in their attitudes about COVID-19, their views on health before and after hospitalization, as well as their experiences of discrimination and harassment. The patients were asked to take the questionnaire home, complete it, and mail it back 1 month after discharge. The questionnaire responses provided data on the basic characteristics of individual patients, changes in their awareness and perceptions of COVID-19, their general outlook after discharge, and incidents of discrimination and harassment.

Given the extent of the COVID-19 outbreak in Japan and the number of cases in our hospital, the study period was divided into two waves: the first wave from March–June 2020 and the second wave from July–September 2020. The patients were grouped according to the time of hospitalization.

#### Details of the questionnaire

Demographics characteristics included the following items: age, sex, residence (whether the patients lived in Tamba City or Tamba Sasayama City), occupation (whether the patients worked in medical or nursing care associated professions), and cohabitants (whether the patients lived alone or with others). We also asked about their reasons for undergoing polymerase chain reaction (PCR) tests (symptomatic patients without close contact with infected individuals, whether they had visited a cluster site, if they had been in close contact with infected individuals, or other reasons). Other data collected included the symptoms on admission, whether they received treatment for COVID-19, and the details, length of hospital stay, and length of hotel stay for isolation. Further, information on any periods when the patients missed work or were unable to perform usual activities of daily living before hospitalization, and the time that passed before they were able to perform usual activities of daily living or resume work after returning home (from the hospital or the quarantine hotel).

The solicited symptoms present at admission included the following: fever, cough, sputum, sore throat, nasal discharge, wheezing, dyspnea, chest pain, myalgia, arthralgia, headache, impaired consciousness, physical lassitude, abdominal pain, nausea, vomiting, diarrhea, taste disorder, and smell disorder. The solicited treatments administered for COVID-19 included the following: antipyretic analgesics, oral medicines for coronavirus, inhalants for coronavirus, oxygen therapy, and mechanical ventilation. Details of the other questions are shown in Additional file 1.

#### Data analysis

Descriptive statistics were used to analyze the patient’s demographics characteristics, the responses to the questions before and after hospitalization for COVID-19 using Stata MP version 15 (StataCorp, College Station, TX, USA).

### Results

During the study period, 39 patients with COVID-19 were discharged from our hospital, and 27 completed questionnaires were received (recovery rate: 69.2%). Table 1 shows the demographics characteristics of patients (missing values were excluded from the calculations), whose mean age was 50.0 ± 17.8 years. Of them, 16 were men (59.3%), while two patients (7.7%) were residents of Tamba City or Tamba Sasayama City (the hospital medical district); the majority of the participants were residents of other medical districts. Nineteen patients (70.4%) were symptomatic.

**Table 1:** Characteristics of study participants who had been hospitalized with COVID-19

	All			1st wave			2nd wave		
	N	n (%)	M (SD)	N	n (%)	M (SD)	N	n (%)	M (SD)
Sex (male)	27	16 (59.3)		16	11 (68.8)		11	5 (45.5)	
Age	27		50.0 (17.8)	16		48.4 (15.2)	11		52.5 (21.6)
Known residence	26			15			11		
Tamba City		0 (0.0)			0 (0.0)			0 (0.0)	
Tamba Sasayama City		2 (7.7)			0 (0.0)			2 (18.2)	
Others		24 (92.3)			15 (100.0)			9 (81.8)	
Occupation (medical or nursing care-associated professions)	24	2 (8.3)		14	2 (14.3)		10	0 (0.0)	
Living alone	25	6 (24.0)		15	2 (13.3)		10	2 (13.3)	
Reasons for undergoing polymerase chain reaction test	26			15			11		
Symptomatic patients without close contact with infected individuals		11 (42.3)			5 (33.3)			6 (54.6)	
Patients who visited a cluster site		2 (7.7)			1 (6.7)			1 (9.1)	
Patients who had a close contact with infected individuals		11 (42.3)			9 (60.0)			2 (18.2)	
Others		2 (7.7)			0 (0.0)			2 (18.2)	
Symptoms present on admission	27	19 (70.4)		27	12 (75.0)		27	7 (63.6)	
Fever	27	12 (44.4)		27	8 (50.0)		27	4 (36.4)	
Cough	27	9 (33.3)		27	5 (31.3)		27	4 (36.4)	
Sputum	27	6 (22.2)		27	2 (12.5)		27	4 (36.4)	
Sore throat	27	4 (14.8)		27	2 (12.5)		27	2 (18.2)	
Nasal discharge	27	1 (3.7)		27	0 (0.0)		27	1 (9.1)	
Wheezing	27	1 (3.7)		27	1 (6.3)		27	0 (0.0)	
Dyspnea	27	4 (14.8)		27	4 (25.0)		27	0 (0.0)	
Chest pain	27	1 (3.7)		27	1 (6.3)		27	0 (0.0)	
Myalgia	27	1 (3.7)		27	1 (6.3)		27	0 (0.0)	
Arthralgia	27	2 (7.4)		27	1 (6.3)		27	1 (9.1)	
Headache	27	7 (25.9)		27	3 (18.8)		27	4 (36.4)	
Impaired consciousness	27	1 (3.7)		27	1 (6.3)		27	0 (0.0)	
Physical lassitude	27	10 (37.0)		27	8 (50.0)		27	2 (18.2)	
Abdominal pain	27	0 (0.0)		27	0 (0.0)		27	0 (0.0)	
Nausea	27	4 (14.8)		27	3 (18.8)		27	1 (9.1)	
Vomiting	27	0 (0.0)		27	0 (0.0)		27	0 (0.0)	
Diarrhea	27	5 (18.5)		27	3 (18.8)		27	2 (18.2)	
Taste disorder	27	7 (25.9)		27	4 (25.0)		27	3 (27.3)	
Smell disorder	27	7 (25.9)		27	4 (25.0)		27	3 (27.3)	
Treatment administered	27	7 (25.9)		27	4 (25.0)		27	3 (27.3)	
Antipyretic analgesics	27	7 (25.9)		27	4 (25.0)		27	3 (27.3)	
Oral medicines for coronavirus	27	0 (0.0)		27	0 (0.0)		27	0 (0.0)	
Inhalants for coronavirus	27	0 (0.0)		27	0 (0.0)		27	0 (0.0)	
Oxygen therapy	27	1 (3.7)		27	1 (6.3)		27	0 (0.0)	
Mechanical ventilation	27	2 (7.4)		27	2 (12.5)		27	0 (0.0)	
Length of hospital stay	27		10.4 (6.8)	16		11.8 (8.3)	11		8.3 (2.8)
Length of hotel stay for isolation	27		5.4 (7.6)	16		8.9 (8.2)	11		0.2 (0.6)
Period when the patients missed work or were unable to perform usual activities of daily living before hospitalization	24		5.8 (7.1)	14		6.1 (5.8)	10		5.4 (9.0)
Time that passed before being able to perform usual activities of daily living or resume work after returning home (from the hospital or the hotel for isolation)	25		9.4 (10.4)	15		10.9 (12.5)	10		7.2 (6.0)

The mean length of hospital stay was 10.4 ± 6.8 days. The period when the patients missed work or were unable to perform usual activities of daily living before hospitalization was 5.8 ± 7.1 days, while the time that passed before being able to perform usual activities of daily living or resume work after returning home was 9.4 ± 10.4 days.

Table 2 shows the responses to the questions regarding perceptions before hospitalization. Before hospitalization, 23 patients (88.5%) feared infection. The percentage of such patients decreased from 100% during the first wave to 70.0% during the second wave. Some measures were taken by 26 patients (96.3%), and social or physical distancing was known by 22 patients (81.5%). The percentage of such patients increased from 68.8% during the first wave to 100.0% during the second wave.

**Table 2:** Questions regarding patients’ disease-prevention awareness prior to being hospitalized for COVID-19

	All		1st wave		2nd wave	
	N	n (%)	N	n (%)	N	n (%)
Were you worried about the risk of infection? (Yes)	26	23 (88.5)	16	16 (100.0)	10	7 (70.0)
Did you take any measures to prevent infection? (Yes)	27	26 (96.3)	16	16 (100.0)	11	10 (90.9)
Did you know about social or physical distancing? (Yes)	27	22 (81.5)	16	11 (68.8)	11	11 (100.0)
Did you place more value on the opinions of celebrities or commentators on TV than on the opinions issued by the government and administration? (Yes)	26	16 (61.5)	16	11 (68.8)	10	5 (50.0)
Did you place more value on the information obtained from the Internet than on the information obtained from TV programs and newspapers? (Yes)	24	8 (33.3)	14	4 (28.6)	10	4 (40.0)

Table 3 shows the responses to the questions regarding life after discharge from the hospital. Most patients (96.2%) followed the recommendations issued by health care centers on recuperation at home. Most patients (96.2%) were treated by their family, colleagues, and friends in the same manner as before hospitalization. However, six patients (23.1%) experienced some sort of discrimination or harassment after discharge (two patients during the first wave [12.5%], and four patients during the second wave [40%]). Two patients (7.7%) experienced discrimination or unreasonable treatment from people around them (no patients during the first wave [0%], two patients during the second wave [20%]). After discharge, 25 patients (96.2%) paid more attention to managing their health than before hospitalization (15 patients during the first wave [93.8%], 10 patients during the second wave [100%]). Hospitalization motivated 20 patients (80%) to change their lifestyles (10 patients during the first wave [66.7%], 10 patients during the second wave [100%]). Six patients (26.1%) thought they would have paid more attention to their lives or the people around them if they had known that they had been infected before they were hospitalized. After discharge, all patients (100%) were more diligent in practicing preventive measures against infection, such as social and physical distancing. After hospital discharge, 19 patients (79.2%) became more willing to take sick leave when they fell ill (nine patients during the first wave [64.3%] and 10 patients during the second wave [100%]).

**Table 3:** Questions regarding the life after discharge following hospitalization for COVID-19

	All		1st wave		2nd wave	
	N	n (%)	N	n (%)	N	n (%)
After discharge, did you follow the recommendations issued by the health care center on recuperation at home? (Yes)	26	25 (96.2)	16	16 (100.0)	10	9 (90.0)
Did you experience any discrimination or harassment? (Yes)	26	6 (23.1)	16	2 (12.5)	10	4 (40.0)
After discharge, did your family, colleagues, and friends treat you in the same manner as before hospitalization? (Yes)	26	25 (96.2)	16	16 (100.0)	10	9 (90.0)
Did you experience discrimination or receive unreasonable treatment from people around you? (Yes)	26	2 (7.7)	16	0 (0.0)	10	2 (20.0)
After discharge, did you think that you could have been sufficiently treated at home without being hospitalized? (Yes)	25	6 (24.0)	16	4 (25.0)	9	2 (22.2)
Do you pay more attention to managing your health since you were discharged than before this hospitalization? (Yes)	26	25 (96.2)	16	15 (93.8)	10	10 (100.0)
Did this hospitalization change your lifestyle? (Yes)	25	20 (80.0)	15	10 (66.7)	10	10 (100.0)
Do you think that you would have given more consideration to your life or people around you if you had known that you had been infected before this hospitalization? (Yes)	23	6 (26.1)	13	3 (23.1)	10	3 (30.0)
Do you practice preventive measures against infection, such as social and physical distancing, more diligently since being discharged? (Yes)	25	25 (100.0)	15	15 (100.0)	10	10 (100.0)
Do you now think that you will be more willing to take a sick leave in the future if you feel sick? (Yes)	24	19 (79.2)	14	9 (64.3)	10	10 (100.0)

### Discussion

The present study revealed that many patients were worried about the risk of infection and took measures against infection before and after hospitalization for COVID-19. It also revealed that they paid more attention to managing their health after discharge. In addition, 23.1% of the patients were found to have experienced some sort of discrimination or harassment after discharge.

Although 23 patients (88.5%) had feared infection before hospitalization, this percentage decreased from 100% during the first wave to 70.0% during the second wave. As the disease outbreak persisted, risk perceptions may have decreased. In Japan, a state of emergency was declared in April 2020 and imposed until mid-May. The measures implemented during the state of emergency included travel restrictions and a ban on late-night business activity for restaurants. The second wave gradually emerged after the state of emergency was lifted. The prolonged outbreak of COVID-19 and lifting the state of emergency might have contributed to the decreased perception of the risk of infection. Furthermore, patients placed more value on the opinions of celebrities or commentators on TV than bulletins issued by the government and administration. Celebrities and commentators appearing on TV should not make careless comments; instead, they should comment after clearly distinguishing facts and plausible conditions from their personal and subjective opinions.

There are studies worldwide describing changes in outlooks, perspectives, and views on health in patients with COVID-19, including experiences with discrimination and harassment [4, 5]. Regarding changes in patients’ outlook, attitudes, and views on health, a study conducted in Bangladesh showed that the overall prevalence of anxiety and depression for COVID patients was 55.7% and 87.3%, respectively [4].

The results revealed that Japanese patients with COVID-19 also changed their views on health. We assume that people changed their views on health after their health and hospitalization circumstances deteriorated due to COVID-19. A February 2020 global survey on discrimination and harassment that included 1904 overseas Chinese residents in 70 countries reported that 25.11% of respondents experienced some form of discrimination, including job dismissal without appropriate reasons or refusal of residency in rental housing [3]. Further, they commonly reported receiving abuse in public places [3]. Another survey of 17,846 people from 31 provinces in mainland China (including autonomous regions, special administrative regions, and cities) found that approximately 90% of respondents indicated that they would report to the authorities if people from the Hubei Province came to their area. Additionally, 50.58% answered that they would avoid people from the Hubei Province, and 16.94% answered that they would actively drive people from the Hubei Province out of their area [3]. Patients with COVID-19 wards face many problems, such as lack of communication and social support and a possible impact on mental health is suggested even in asymptomatic patients [5]. Changes in views on health have been associated with anxiety and depression, and the incidents of some form of discrimination and harassment resemble the cases reported in the Hubei Province of China. Thus, Japan is no exception; the present study revealed the presence of patients who experienced some sort of discrimination or harassment. It is not clear what the underlying causes of the discrimination or harassment in the current study. Japan is a fairly homogeneous society and there are cases of discriminations against minorities and foreign residents [6]. Also social ostracism is a deep history in Japan [6]. Patients with COVID-19 may infect others and neighbors may have thought causing trouble to others is irresponsible and a great shame.

Further, the results indicated that the patients recognized the negative effect of presenteeism and thought that they should take leave when they felt sick. While there are variations in the definition of presenteeism, related to employees’ health, it is the phenomenon of going to work even when it would be better to take sick leave and rest because of poor health [7]. A wide range of personal, organizational, and occupational factors are known to affect decisions on whether to work or take sick leave [8]. Personal factors include unwillingness to burden colleagues [9], a strong sense of professionalism [10], the inability to say no, and low income [11]. Occupational factors include lack of employees [11], a benefit program that discourages employees from taking sick leave [12], poor leadership [13], and instability of employment [14]. It is also suggested that such behaviors of physicians may contribute to the spread of infection [15].

The effective management of presenteeism is a long-term investment for the well-being of people and organizations. It is important, particularly during a pandemic, to allow employees to remain healthy and perform at their best. Now seems to be the best time to deliver this message to society.

### Conclusion

Although patients with COVID-19 had a strong fear of infection before hospitalization, they became more conscious of their health after discharge. For example, they paid more attention to managing their health and became more willing to take sick leave if they become ill. In addition, as many as 23.1% of the patients with COVID-19 experienced discrimination or harassment. Proactively addressing harassment and discrimination through campaigns in workplaces and public health announcements and distributing factual information about risks may reduce the occurrence of this treatment of people in future health crises.

## Limitations

The present study was conducted at a single center in Japan during the relatively early stages of the COVID-19 pandemic. The generalization of its results requires caution because of the small sample size and the fact that the study was conducted early in the pandemic.

## Supplementary Information

**Additional file 1.** Questions asked to investigate the patients perceptions before hospitalization and after discharge from hospital for COVID-19.

## Data Availability

The datasets used and/or analyzed during the current study are available from the corresponding author on reasonable request.
