# Supplementary material for: Changes in patients’ outlook, behaviors, and attitudes toward COVID-19 after hospitalization and their experiences of discrimination and harassment
Source: BMC Res Notes. 2021 Sep 17;14:362. doi: 10.1186/s13104-021-05780-9 (PMC8447803; doi:10.1186/s13104-021-05780-9)
Supplement: Supplementary file 1 — Additional file 1. Questions asked to investigate the patients perceptions before hospitalization and after discharge from hospital for COVID-19. [file 13104_2021_5780_MOESM1_ESM.docx]

The following binary-choice questions (yes/no) were asked to investigate the patients’ perceptions before hospitalization for COVID-19:

Question 1: Were you worried about the risk of infection?

Question 2: Did you take any measures to prevent infection?

Question 3: Did you know about social or physical distancing?

Question 4: Did you value the opinions of celebrities or commentators on TV more than the opinions issued by the government and administration?

Question 5: Did you value the information obtained from the Internet more than the information obtained from TV programs and newspapers?

The following binary-choice questions (yes/no) were asked to investigate the lives of the patients after discharge from hospital for COVID-19:

Question 1: After discharge, did you follow the recommendations issued by the health care center on recuperation at home?

Question 2: Did you experience any discrimination or harassment?

Question 3: After discharge, did your family, colleagues, and friends treat you in the same manner as before hospitalization?

Question 4: Did you experience discrimination or unreasonable treatment from people around you?

Question 5: Did you think, after discharge, that you could have been sufficiently treated at home without being hospitalized?

Question 6: Do you pay more attention to managing your health after discharge than before this hospitalization?

Question 7: Did this hospitalization change your lifestyle?

Question 8: Do you think that you would have given more consideration to your life or people around you if you had known that you had been infected before this hospitalization?

Question 9: Do you practice preventive measures against infection, such as social and physical distancing, more diligently after discharge?

Question 10: Do you now think that you will be more willing to take sick leave if you feel ill?
